# REC, *Drosophila* MCM8, Drives Formation of Meiotic Crossovers

**DOI:** 10.1371/journal.pgen.0010040

**Published:** 2005-09-23

**Authors:** Hunter L Blanton, Sarah J Radford, Susan McMahan, Hutton M Kearney, Joseph G Ibrahim, Jeff Sekelsky

**Affiliations:** 1 Curriculum in Genetics and Molecular Biology, University of North Carolina, Chapel Hill, North Carolina, United States of America; 2 Program in Molecular Biology and Biotechnology, University of North Carolina, Chapel Hill, North Carolina, United States of America; 3 Department of Biology, University of North Carolina, Chapel Hill, North Carolina, United States of America; 4 Department of Biostatistics, University of North Carolina, Chapel Hill, North Carolina, United States of America; Stowers Institute for Medical Research, United States of America

## Abstract

Crossovers ensure the accurate segregation of homologous chromosomes from one another during meiosis. Here, we describe the identity and function of the *Drosophila melanogaster* gene *recombination defective (rec),* which is required for most meiotic crossing over. We show that *rec* encodes a member of the mini-chromosome maintenance (MCM) protein family. Six MCM proteins (MCM2–7) are essential for DNA replication and are found in all eukaryotes. REC is the *Drosophila* ortholog of the recently identified seventh member of this family, MCM8. Our phylogenetic analysis reveals the existence of yet another family member, MCM9, and shows that MCM8 and MCM9 arose early in eukaryotic evolution, though one or both have been lost in multiple eukaryotic lineages. *Drosophila* has lost MCM9 but retained MCM8, represented by REC. We used genetic and molecular methods to study the function of REC in meiotic recombination. Epistasis experiments suggest that REC acts after the Rad51 ortholog SPN-A but before the endonuclease MEI-9. Although crossovers are reduced by 95% in *rec* mutants, the frequency of noncrossover gene conversion is significantly increased. Interestingly, gene conversion tracts in *rec* mutants are about half the length of tracts in wild-type flies. To account for these phenotypes, we propose that REC facilitates repair synthesis during meiotic recombination. In the absence of REC, synthesis does not proceed far enough to allow formation of an intermediate that can give rise to crossovers, and recombination proceeds via synthesis-dependent strand annealing to generate only noncrossover products.

## Synopsis

Most of our cells have two copies of each chromosome. For sexual reproduction, these must separate from one another to produce sperm or eggs with one copy of each chromosome. This occurs during meiosis, when chromosomes pair and exchange DNA segments. This exchange— meiotic recombination—creates physical linkages between chromosome pairs and is also a source of genetic diversity. To learn more about the process of meiotic recombination, the authors characterized the gene *recombination defective (rec)* from the fruit fly *Drosophila melanogaster*. Molecular analysis revealed that *rec* is related to a large family of genes found in all animals, plants, and protists. These genes are thought to be important in DNA replication, but *rec* appears to have a novel function. The authors found that mutants lacking *rec* are unable to copy enough DNA during meiotic recombination to form linkages between chromosomes. This results in chromosomes segregating randomly during meiosis, so that most eggs have an incorrect number or composition of chromosomes.

## Introduction

Faithful segregation of homologous chromosomes in meiosis requires crossovers, which, in concert with sister chromatid cohesion, form the chiasmata that hold and orient homologs on the meiotic spindle. Crossovers are distributed nonrandomly between chromosomes, along each chromosome arm, and relative to one another, indicating that meiotic recombination is tightly regulated. One aspect of this regulation is the process that determines whether a recombination event becomes a crossover or a noncrossover.

**Figure 1:**
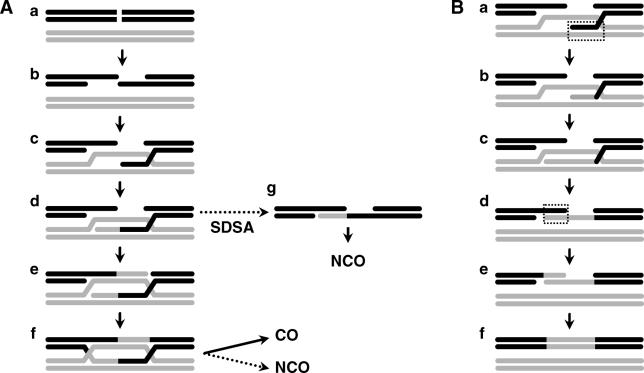
Meiotic Recombination Models (A) Model for the formation of crossovers (CO) and noncrossovers (NCO). Recombination is initiated with a DSB (a), that is resected to generate 3′ single-stranded ends (b). A 3′ end invades the homologous chromosome (c) and primes repair DNA synthesis (d). If repair synthesis proceeds far enough, second-end capture can occur (e). After repair synthesis from the second end, ligation of newly synthesized 3′ ends to resected 5′ ends results in a DHJ intermediate (f). Resolution of the DHJ can generate a CO or a NCO. In SDSA, the nascent strand dissociates after repair synthesis and anneals to the second broken end (g). Gap filling and ligation generates an NCO. Note that NCO products can come from either a DHJ intermediate or SDSA, or both. (B) Sources of gene conversion. hDNA is first generated during strand invasion (a, dotted box); mismatch repair may replace the invading sequence with the sequence of the template (b). After synthesis (c) and dissociation, annealing of the nascent strand to the other end of the break also generates hDNA (d), which can also be acted on by mismatch repair (e). The final NCO product in this example (f) has a region of gene conversion derived from two rounds of mismatch repair, but one round is sufficient to generate a gene conversion. Also, mismatch repair can act on the DHJ intermediate (A) or the products of DHJ resolution, and thus gene conversion can also be associated with crossing over.

Models of meiotic recombination must account for the production of both crossovers and noncrossovers. Current models are derived from the double-strand break (DSB) repair model of Szostak et al. [1]. In this model, recombination is initiated with a DSB on one chromatid (Figure 1A, parts a–f). Resection of the 5′ ends leaves 3′ single-stranded overhangs. One of these overhanging ends invades a homologous, non-sister duplex and primes repair DNA synthesis. The strand displaced by the migrating synthesis bubble is captured by the other 3′ overhang, which primes synthesis using the displaced strand as a template. Ligation of the newly synthesized ends to the resected 5′ ends generates an intermediate with two Holliday junctions. This double Holliday junction (DHJ) intermediate is resolved by an unknown endonuclease to form either crossover or noncrossover products.

Recent data from yeast has resulted in modification of this model. Allers and Lichten [2] physically monitored formation of recombination intermediates and products in *Saccharomyces cerevisiae* using an ectopic recombination system and found that noncrossover products appear before DHJ intermediates. They proposed that noncrossovers arise not through a DHJ intermediate, but through synthesis-dependent strand annealing (SDSA). In SDSA, the nascent strand dissociates from the template and anneals to the other resected end (Figure 1A, part g). Trimming of any overhangs and filling in of any gaps, followed by ligation, results in noncrossover products. Subsequent genetic tests of this model in *S. cerevisiae* are consistent with most noncrossovers coming from SDSA, while the remainder are derived from a DHJ intermediate [3].

These models also take into account the occurrence of gene conversion—nonreciprocal transfer of information from one duplex to another—that can be associated with both crossovers and noncrossovers. Figure 1B illustrates possible origins of gene conversion during SDSA. Heteroduplex DNA (hDNA), in which the two strands are derived from different parental molecules, is produced by both invasion of a single-stranded overhang into a homologous template and annealing of a newly synthesized strand to the other single-stranded overhang. Sequence differences between homologous chromosomes result in base/base mismatches and insertion/deletion heterologies in hDNA, and these can be recognized and repaired by the mismatch repair system. The product contains a region of sequence derived from the homologous chromosome, referred to as a gene conversion tract. If heterologies are not repaired, each strand will convey different genetic information to the haploid product of meiosis. Upon the first round of DNA replication and mitosis after fertilization or germination, these strands separate, resulting in the post-meiotic segregation (PMS) of parental alleles. PMS results in a mosaic individual, or, for unicellular eukaryotes, a sectored colony.

Though it is more difficult to physically observe intermediates formed during meiotic recombination in *Drosophila,* a wealth of evidence indicates that recombination is also initiated by DSBs in this organism. MEI-W68, the *Drosophila* ortholog of Spo11, which catalyzes meiotic DSB formation in *S. cerevisiae,* is required to generate both crossovers and noncrossovers, and in *mei-W68* mutants recombination is restored by treatment with ionizing radiation [4,5]. Mutations in *Drosophila* genes required for strand invasion cause female sterility that is suppressed by mutation of *mei-W68* [6–10]. Thus, the early steps in meiotic recombination appear to be similar between *Drosophila* and *S. cerevisiae*. In contrast, later stages of crossover production are different, since most crossovers in *Drosophila* require the XPF/Rad1 ortholog MEI-9 [10,11], its binding partner ERCC1 [12], and several novel proteins, including MUS-312 [13] and MEI-218 [14]. In addition, it is not known whether noncrossovers in *Drosophila* are derived from a DHJ intermediate or SDSA, although SDSA is the most common pathway for repair of mitotic DSBs generated by transposable element excision [15–17].

In *Drosophila,* mutagenesis screens have been used to identify many novel genes required for meiotic recombination. The gene *recombination defective (rec)* was identified more than 25 years ago by Rhoda Grell in an ethyl methanesulfonate (EMS) screen for temperature-sensitive recombination mutants [18]. Her preliminary characterization of two null alleles showed that *rec* mutants have high levels of chromosome nondisjunction and reduced fertility, both indicative of homologous chromosome segregation defects. Since these mutants are able to pair homologous chromosomes normally, but exhibit a severe reduction in crossing over, Grell concluded that *rec* is involved in generating meiotic crossovers.

To gain insight into the function of the REC protein in meiotic recombination, we identified *rec* molecularly and found that it encodes the *Drosophila* ortholog of MCM8. The eukaryotic mini-chromosome maintenance (MCM) family of proteins contains six members (MCM2–7) that form a heterohexameric helicase required for replication [19]. Though MCM2–7 are essential in all eukaryotes, *rec* mutants are viable, and we have been unable to find any function for REC outside of meiosis. To explore the defect in meiotic recombination further, we analyzed the distribution of crossing over in *rec* mutants and found that residual crossovers are distributed abnormally. This finding, coupled with epistasis analysis, suggests that REC might act at an intermediate step in recombination. Further insight into the function of REC comes from our finding that the frequency of noncrossovers is substantially increased in *rec* mutants, and that these noncrossover events have significantly shorter gene conversion tracts than those of wild-type females. Based on these phenotypes and the structural similarity between REC and MCM proteins, we propose that REC facilitates processive repair DNA synthesis, and is a prerequisite for formation of the DHJ intermediate during meiotic recombination. In the absence of REC, recombination proceeds through SDSA to generate noncrossovers.

## Results

### Molecular Identification of *rec*

Grell's early work on the null alleles of *rec* found that homozygous mutant females produce progeny with high levels of chromosome nondisjunction [18]. Elevated levels of nondisjunction are indicative of, among other things, defects in homolog synapsis or meiotic recombination. Grell found that although synaptonemal complex formation was normal in *rec* mutant females, the frequency of crossing over among progeny recovered was 3.6% of wild-type levels. This crossover reduction indicates that the product of *rec* is involved in the meiotic recombination pathway and is required to generate most crossovers. To understand the function of REC in generating crossovers, we first sought to identify the gene molecularly.

**Figure 2:**
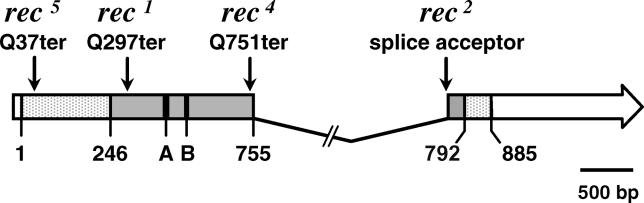
Mutations in *rec* The *rec* gene contains two exons and an intron. Untranslated regions are open and protein-coding regions are filled, with the MCM core indicated by gray fill. Amino acid numbers are shown below the model, and the positions of the four mutations sequenced are shown above. The locations of the Walker A and B boxes are shown as black bars and are labeled below the model. Only the position of the intron is indicated. The full intron is 5.5 kilobases and contains another predicted gene *(CG4576),* transcribed in the opposite direction.

Matsubayashi and Yamamoto [20] used deletion and restriction mapping to place *rec* in a region on the right arm of Chromosome 3 between the genes *c(3)G* and *spn-E*. Subsequently, *c(3)G* and *spn-E* were molecularly identified and the region was sequenced, revealing nine predicted genes in the region to which *rec* was mapped. We identified *CG31293* as a candidate for *rec* because it was the only gene in the region that encodes a protein whose proposed function is associated with DNA metabolism. *CG31293* has two exons separated by a 6-kilobase intron. We sequenced *CG31293* in *rec^1^* and *rec^2^* mutant flies and found mutations in both (Figure 2). Sequencing *CG31293* in *rec^1^* homozygotes revealed a C→T transition at position 889, which changes a glutamine codon to a stop codon. If translated, this mutation would result in a truncation after 295 of 885 amino acids. The *rec^2^* allele has an A→T transversion that disrupts the *CG31293* splice acceptor site.

To generate additional alleles of *rec,* we screened the progeny of females with an EMS-mutagenized Chromosome 3 in *trans* to *rec^2^* for high levels of X chromosome nondisjunction (see Materials and Methods for details). Out of 1,238 lines screened, we obtained two alleles of *rec,* both of which have mutations in *CG31293*. The *rec^4^* allele has a C→T transition at nucleotide 2251 that generated a nonsense codon near the end of the first exon; *rec^4^* mutants could potentially produce a protein of the same approximate size as the *rec^2^* mutant. The *rec^5^* allele has a C→T transition at nucleotide 142 that generated a nonsense codon predicted to truncate the protein after only 47 residues. Based on the sequence of these four alleles, we conclude that *rec* corresponds to *CG31293* and that all four alleles are likely to be null.

During the course of this study, Matsubayashi and Yamamoto, using a different strategy also concluded that *rec* corresponds to *CG31293* [21]. Their sequence analysis of *rec^1^* and *rec^2^* corresponds with our findings. They also sequenced the temperature-sensitive allele *rec^3^* and found a C→T transition at nucleotide 1379, which causes the substitution of a nonconserved serine for a phenylalanine at residue 455.

### MCM8 and MCM9 Arose Early in Eukaryotic Evolution

*CG31293* encodes an 885–amino acid protein that is homologous to the MCM family of proteins found throughout eukaryotes and archaea [19]. Archaeal species each have a single MCM that forms a homohexamer believed to be the replicative helicase. Eukaryotes have six related MCM proteins, MCM2–7, which constitute a hexameric helicase involved in replication, recombination, and transcription [22]. REC is the *Drosophila* ortholog of MCM8, a seventh eukaryotic member of this family that was identified recently [21,23–26]. MCM8 has been reported to be present in vertebrates and *Drosophila,* but not in fungi or nematodes, and thus the origin of this family member is unclear.

To learn more about the MCM family of proteins, we analyzed MCMs from a number of diverse eukaryotes. On the basis of molecular and ultrastructural analysis, eukaryotes can be divided into eight major phylogenetic groups [27]. Previously, MCMs have been analyzed from only two of these groups: plants, represented by *Arabidopsis thaliana,* and opisthokonts, which include fungi and animals. In addition, Gozuacik et al*.* [26] noted the existence of an MCM8 ortholog in the discicristate *Leishmania major,* suggesting a broad phylogenetic distribution of this family member among eukaryotes. To expand this analysis, we identified all of the MCMs from species for which complete or nearly complete genome sequence is available, including at least one from six of the eight eukaryotic phylogenetic groups (Table S1). We conducted alignments on the predicted protein sequence of each conserved MCM domain and used these alignments to construct phylogenetic trees (see Materials and Methods for details). Eukaryotic MCM domain proteins cluster into eight subfamilies comprising the six replicative MCMs, MCM8, and a novel group that we refer to as MCM9 (Figure 2). Classification in these eight subfamilies was the same with two different algorithms for tree construction (maximum likelihood and neighbor joining), although the relationship of each group to one another and group members to each other varies with different methods of tree construction.

As expected, the six replicative MCMs are present in all eukaryotes, indicating that these six were present in the ancestral eukaryote. MCM8 and MCM9 are also widely distributed among eukaryotes, found in five of the six phylogenetic groups we examined, suggesting that the MCM8 and MCM9 subfamilies also arose early in eukaryotic evolution. Because both are absent from the excavate *Giardia lamblia,* which is perhaps the most deeply branching species surveyed, we cannot determine whether these family members originated after a split between excavates and other eukaryotes, or whether they were lost in the lineage giving rise to *Giardia*. MCM8 and MCM9 are also absent from the nematode *Caenorhabditis elegans* and from all available fungal genomes, with the exception of the microsporidium *Encephalitozoon cuniculi*. Thus, MCM8 and MCM9 do appear to have been lost multiple times during the eukaryotic radiation. Interestingly, our survey suggests that in most eukaryotic genomes MCM8 and MCM9 are either both present or both lacking. This raises the possibility that these two family members function together.

Mammalian MCM9 is unique in that it lacks the carboxy-terminal half of the conserved MCM domain, including the Walker B box (Figure S1). This is unlikely to be an error in annotation, since all mammalian MCM9 sequences available have a similar structure, and several full-length cDNA sequences encoding the truncated human protein are present in the database. Although mammalian MCM9 would lack ATPase activity on its own, it is possible that this protein retains some other function.

**Figure 3:**
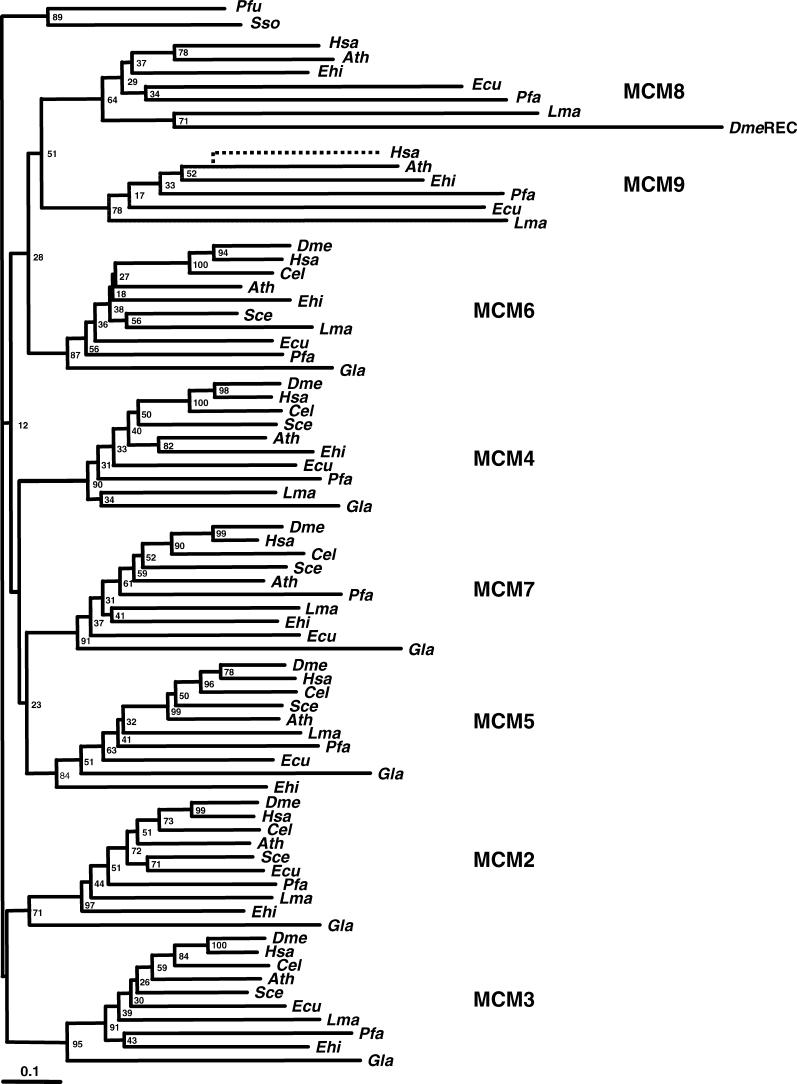
Phylogenetic Analysis of Eukaryotic MCM Family Proteins The tree shown was generated by the neighbor-joining method of ClustalW, using an alignment of core MCM domains, excluding positions with gaps and correcting for multiple substitutions. The numbers on each node are the percentage of trees with the given branch from 10,000 independent boot-strapped iterations. Human MCM9 was excluded from this analysis because it lacks the carboxy-terminal half of the MCM domain. The approximate position of this protein was inferred from other analyses and is indicated with a dotted line. This tree was rooted with a branch containing two archaeal MCMs. The scale represents the relationship of branch length to phylogenetic distance expressed as the number of substitutions per site. Organisms are abbreviated as follows: *Pfu, Pyrococcus furiosum; Sso, Sulpholobus sulfataricus; Hsa, Homo sapiens; Dme, Drosophila melanogaster; Cel, Caenorhabditis elegans; Sce, Saccharomyces cerevisiae; Ecu, Encephalitozoon cuniculi; Ath, Arabidopsis thaliana; Ehi, Entamoeba histolytica; Pfa, Plasmodium falciparum; Lma, Leishmania major; Gla, Giardia lamblia*.

Three features of the phylogenetic tree in Figure 3 indicate that *Drosophila* REC has diverged from other MCM8 proteins. First, REC never clusters adjacent to human MCM8, despite the fact that this is the most closely related of the species shown (compare clustering of replicative MCMs). Second, *Drosophila melanogaster* is the only species surveyed for this figure that has MCM8 but lacks MCM9. This is true for other *Drosophila* species, including *D. pseudoobscura* and *D. virilis,* which diverged from *D. melanogaster* approximately 25 and 40 million years ago, respectively (Figure S2). However, another arthropod, the mosquito *Anopheles gambiae,* has retained both MCM8 and MCM9.

The third notable feature of REC is that it has a longer branch length than other MCMs, indicating that it has accumulated more substitutions than its orthologs. Alignment of MCMs from several diverse eukaryotes and several *Drosophila* species (Figure S2) shows some significant differences between REC and other MCMs. Replicative MCMs have consensus sequences for the Walker A and B boxes, which are involved in nucleotide binding and hydrolysis [28], and differ from those of other ATPases. The A box consensus sequence of GxxGxGKT is GDP[GS]x[SA]KS (x represents any residue; residues in brackets are alternative possibilities at a given site) in replicative MCMs. Although other MCM8 and MCM9 sequences match the replicative MCM consensus, REC has a sequence closer to the canonical ATPase consensus: GDPGIGKT. Replicative MCMs also have a highly conserved Walker B signature of IDEFDKM. Even within the deeply branching protists, the only substitutions are replacement of the initial isoleucine with a different bulky aliphatic residue (L, V, or M), replacement of the central phenyalanine with a different hydrophobic residue (leucine in several *G. lamblia* MCMs), or replacement of the second aspartic acid residue with glutamic acid. The corresponding sequence of REC, however, is LDDVDKL, which has five substitutions in the seven-residue sequence. However, each substitution represents a conservative change, suggesting that ATPase function may have been conserved. We also note that the DK residues in this motif are not conserved in the MCM9 subfamily. A third highly conserved motif found in MCM proteins is the arginine finger found downstream of the Walker B box. The consensus for this motif is UUSRFDUU, where U is a bulky aliphatic residue (I, L, V, or M). This motif is found in all eukaryotic and archaeal MCMs surveyed except *Arabidopsis* MCM9 and *Drosophila* REC. In REC, this sequence is LLREFHLV, and thus the absolutely conserved SR and D residues are missing.

In spite of the important sequence differences between REC and other MCMs, it is clear that REC is a member of the MCM family. When REC is used as a query in BLAST searches, all MCM proteins are returned with a high score, while other sequences do not receive significant scores. In addition, searches for conserved domains identify the MCM domain with high confidence. The divergence of *Drosophila* REC from other MCMs may be related to the absence of MCM9 in *Drosophila,* perhaps by allowing REC to assume functions of both proteins. Alternatively, REC may have acquired a novel function that does not require MCM9.

Though REC has sequence homology to replicative MCMs, *rec* mutants do not have phenotypes consistent with an essential role in replication. The genes encoding *Drosophila* MCM2–7 are essential [29–33], but *rec* mutants are viable and do not exhibit any of the gross developmental defects observed in the replicative MCM mutants. However, *rec* is expressed in somatic tissues—cDNAs have been identified in libraries made from embryos, adult head, and cell lines [34]—suggesting a function outside of meiosis. Since many of the genes required for meiotic recombination are also required for mitotic repair of DNA damage [35], we hypothesized that REC may be involved in repair of DNA damage in somatic cells. To test this hypothesis, we measured the sensitivity of *rec* mutants to agents that cause DSBs (gamma irradiation) and block replication (methyl methanesulfonate [36] and nitrogen mustard), following previously published methods [37,38]. We found that *rec* mutants are not hypersensitive to either of these agents when compared to their heterozygous siblings (unpublished data). We conclude that REC is not essential for repair of DSBs or blocked replication forks in mitotically dividing cells. Matsubayashi and Yamamoto also found that *rec* mutants are not hypersensitive to X-rays or methyl methanesulfonate [21].

### REC Functions in an Intermediate Step of Meiotic Recombination

The only known function for REC is in generating meiotic crossovers. As a putative replicative helicase, there are many steps in recombination where REC might act to promote crossing over, including pre-meiotic S phase, resection, strand invasion, repair synthesis, and resolution. Because *rec* mutants have normal synaptonemal complex formation [18], it is unlikely that REC is required to complete pre-meiotic S phase. To determine where REC acts in subsequent steps of meiotic recombination, we first placed *rec* genetically in the *Drosophila* recombination pathway relative to previously characterized genes. To accomplish this, we conducted epistasis studies and analyzed the distribution of residual crossovers in *rec* mutants. In *Drosophila,* null mutations in genes whose products are required to generate meiosis-specific DSBs *(mei-W68, mei-P22)* abolish essentially all crossovers [4]. Because null *rec* mutants have residual crossovers, we conclude that REC is not involved in DSB formation like MEI-W68 and MEI-P22. After DSB formation, broken ends are resected and Rad51 homologs catalyze strand invasion. Females mutant for *spn-A,* which encodes the *Drosophila* ortholog of Rad51, are sterile and lay eggs with developmental patterning defects [9]. These phenotypes are due to an activated DNA damage-dependent cell-cycle checkpoint caused by persistent unrepaired meiotic DSBs, which disrupts communication between the oocyte and the somatic follicle cells that pattern the eggshell [10]. The proteins required for resection are not known, but we expect *Drosophila* resection mutants to have a phenotype similar to *spn-A* mutants because they would also be unable to repair meiotic DSBs. Because *rec* mutants are not sterile and have normal eggshell patterning, it is unlikely that REC is required for resection or strand invasion. To determine whether REC functions before or after strand invasion, we generated *rec spn-A* double mutants. The phenotype of the double mutant females was similar to that of *spn-A* single mutants, including sterility and patterning defects (unpublished data). Though this does not exclude the possibility that REC could be acting in an accessory role at either resection or strand invasion, it suggests that REC acts after strand invasion.

Further insight into the function of REC can be gained by examining the distribution of residual crossovers in *rec* mutants. In wild-type females, most crossovers are located in the middle of a chromosome arm and very few occur near centromeres or telomeres. In most recombination mutants that do not completely abolish crossing over, the reduction in crossing over is polar, with a stronger reduction in medial intervals and less reduction in intervals proximal to the centromere [39,40]. Null mutations of *mei-218* and hypomorphic alleles of genes required for DSB formation and strand invasion have this phenotype [41]. The only known recombination mutants that do not have a polar reduction are *mei-9,* which encodes the *Drosophila* ortholog of the endonuclease XPF, and *mus312,* which encodes a protein required for the meiotic function of MEI-9 [13,39]. MEI-9 and MUS312 are believed to act together in the final steps of the crossover pathway, perhaps in resolution of DHJ intermediates. Resolution of Holliday junctions in *Escherichia coli* requires the branch migration activity of the helicase RuvB (reviewed in [42]), and in mammalian cells Holliday junction branch-migration activity co-purifies with resolvase [43]. If REC has helicase activity that is required for resolution, we would expect *rec* mutants to exhibit a uniform reduction in crossing over, as in *mei-9* mutants.

To examine crossover distribution in *rec* mutants, we measured the frequency of recombination on Chromosome 2 using intervals flanked by visible markers that span the entire left arm and centric heterochromatin. We calculated the ratio between the reduction in crossover frequency in the centromere-proximal interval *(pr-cn)* and the reduction across the entire arm *(al-cn)* (Table 1)*.* In *mei-9* mutants this ratio was 1.1, showing that the reduction in crossing over is the same in the centromere-proximal interval as in other regions of the chromosome. In *mei-218* mutants, however, this ratio was 4.0, due to a more modest reduction in the interval proximal to the centromere. When we analyzed the frequency and distribution of crossovers in *rec* mutants, we found a more modest reduction in crossing over in the centromere-proximal interval compared to the total crossover reduction (*pr-cn* to *al-cn* ratio of 3.3). Based on this analysis, we conclude that REC does not act with MEI-9 at the resolution step of meiotic recombination.

**Table 1:**
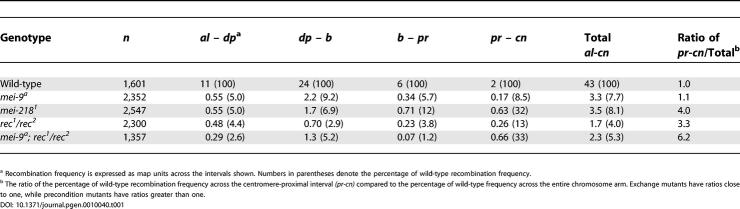
Crossing Over on the Second Chromosome in Recombination Mutants

Because *mei-9* and *rec* have different distributions of residual crossovers, we were able to perform epistasis analysis between these two genes. As in the *rec* single mutant, the reduction in crossing over is polar in the double mutant, with a *pr-cn* to *al-cn* ratio of 6.2. Although this ratio is actually higher than that of the *rec* single mutant, it is unclear whether this difference is functionally significant or is merely an anomaly caused by genetic background effects or relatively low sample size. (Due to the difficulty of the genetic manipulations involved in generating and assaying the double mutant, we were not able to generate as large a sample size for this genotype as for the single mutants; for example, we recovered only a single crossover within the *b-pr* region, resulting in an apparently more severe decrease in this region than in other regions, though recovery of one additional crossover would have made this region appear more like the *al-dp* region.) Regardless of the reason for the differences between the double mutant and the *rec* single mutant, it is clear that the double mutant exhibits a polar phenotype, confirming that REC does not function at the same time as MEI-9 but most likely acts at an intermediate step in the recombination pathway.

### *rec* Mutants Have Increased Rates of Noncrossover Recombination but Shorter Gene Conversion Tracts

Our data place REC in an intermediate step of the recombination pathway, sometime after strand invasion. These steps include repair DNA synthesis, capture of the second resected end, and ligation to form a DHJ intermediate. Because REC is homologous to MCM replication proteins, we hypothesized that REC acts during repair DNA synthesis. Repair synthesis is necessary for formation of both crossovers and noncrossover gene conversions. If REC is essential for all repair synthesis during meiosis, then *rec* mutants should exhibit a reduction in the frequency of noncrossovers similar to the reduction observed in crossovers. Alternatively, if REC merely facilitates repair synthesis, then there may be no reduction in the frequency of noncrossovers in *rec* mutants, but gene conversion tracts should be shorter than in wild-type.

**Figure 4:**
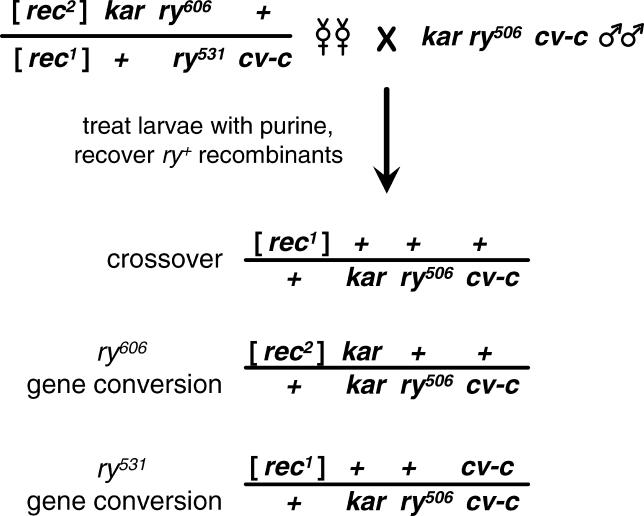
Intragenic Recombination at *rosy* Cross scheme used to recover recombination events within the *ry* gene. Brackets indicate that females were either *trans*-heterozygous for different *rec* alleles or were completely wild-type at *rec*. The three types of *ry+* progeny recovered after purine selection are indicated.

To determine the frequency of noncrossovers and the length of conversion tracts, we recovered noncrossover gene conversions. Because there are no hotspots for recombination in *Drosophila,* we used a system originally developed by Chovnick and colleagues to select for recombination events that occur at the *rosy (ry)* locus [44,45]. The *ry* gene encodes xanthine dehydrogenase, an enzyme that metabolizes purine and is required for normal eye pigmentation; *ry* mutant larvae die when the food is supplemented with purine. Females *trans*-heterozygous for *ry^531^* and *ry^606^*, missense mutations separated by 3.8 kilobases, are crossed to males that are homozygous for a deletion of *ry* (Figure 4). The progeny are treated with purine so that only rare *ry^+^* recombinants and mosaics that have both *ry^+^* and *ry* mutant tissue (products of PMS) survive. Visible markers flanking *ry (kar* and *cv-c)* were used to distinguish crossovers from noncrossover recombination events.

We screened more than 2.3 million progeny of wild-type females and more than 0.7 million progeny of *rec* mutant females (Table 2). As expected, the frequency of crossing over in *rec* mutants was less than 10% of the wild-type frequency. In contrast, noncrossover gene conversions were recovered almost twice as frequently in *rec* mutants as in wild-type. Increased recovery of noncrossovers in *rec* mutants could reflect an actual increase in the frequency of events, or it could be caused by an increase in gene conversion tract length. An increase in tract length increases the frequency of recovery of recombination events because selecting for *ry^+^* recombinants enriches for longer conversion tracts. Since there are no recombination hotspots in *Drosophila,* recombination could be initiated at any site within or near *ry* [46]. Therefore, the longer a conversion tract is, the greater the probability that it crosses either the *ry^606^* or *ry^531^* mutant site. (There is also a selection against extremely long tracts, because these might span both the *ry^606^* and *ry^531^* sites and convert the mutant site to wild-type and the wild-type site to mutant, producing a mutant allele of *ry*. However, this effect is negligible due to the relatively large distance separating the two mutant sites [47].)

**Table 2:**
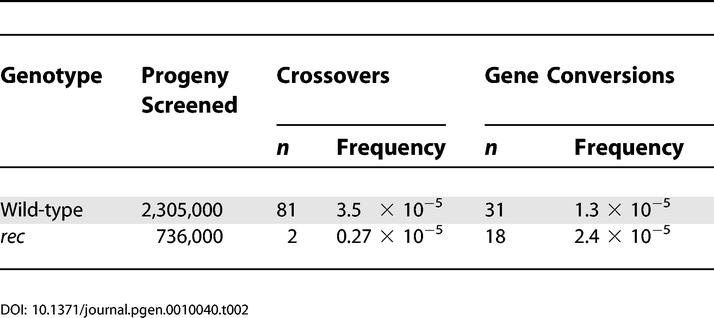
Intragenic Recombination in Wild-Type and *rec* Mutants

**Figure 5:**
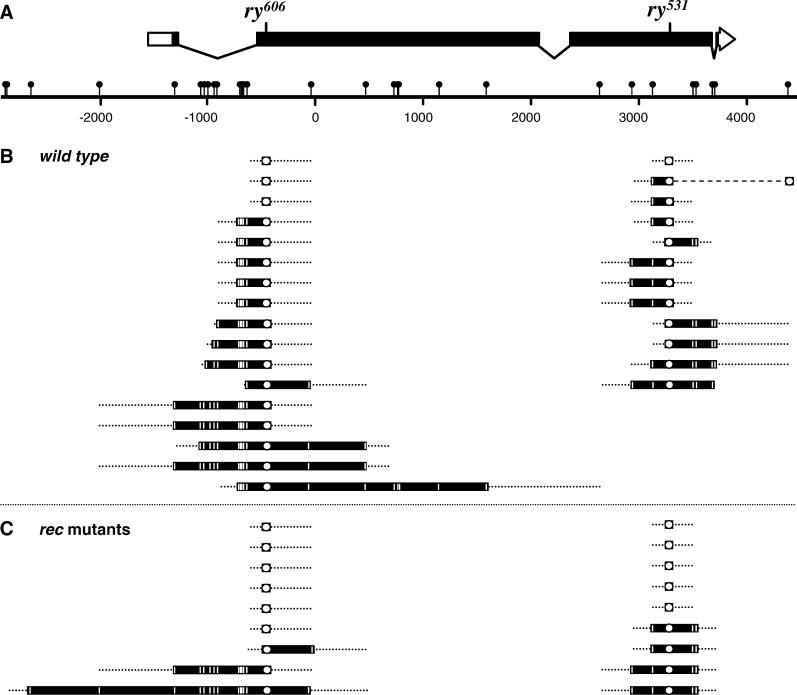
Gene Conversion Tracts from Wild-Type and *rec* Mutants (A) Schematic of the *rosy* locus. Intron/exon structure is shown, with coding sequences filled. The positions of the selected sites corresponding to the *ry^606^* and *ry^531^* mutations are shown. Heterologies between the *ry^606^* and *ry^531^* chromosomes are indicated as lollipops on the scale bar. These are all single nucleotide polymorphisms, except for −1029 and −685, which are insertions of one- and four-bp, respectively, in *ry^531^* relative to *ry^606^*. The scale is in bp, using the coordinate system of Bender et al. [56]. (B and C) Tract lengths observed in NCOs recovered from wild-type (B) and *rec* mutants (C). Each bar represents an independent event, with the open circle denoting the selected marker (*ry^606^* or *ry^531^* mutant sites). Black bars represent the minimum tract length for each event, with co-converted sites marked by white lines. Dotted lines represent the maximum tract length possible based on the next unconverted polymorphism. The dashed line in the second *ry^531^* conversion on panel B indicates a possible discontinuous conversion tract.

Because we did not observe a reduction in the frequency of noncrossovers, we can conclude that REC is not essential for all repair synthesis during meiosis. To test our hypothesis that REC facilitates repair synthesis, and to determine why we recovered more noncrossover gene conversions in *rec* mutants, we measured gene conversion tract lengths in the events recovered. We were able to map gene conversion tracts because the *ry* chromosomes we used are polymorphic across their entire length. We mapped 33 single nucleotide polymorphisms, or small insertion/deletion heterologies, within a 7.3-kilobase region that includes the *ry^531^* and *ry^606^* sites (Figure 5A and Table S2). In contrast to the case in fungi, this level of polymorphism (~0.5%) has no effect on recombination frequency in *Drosophila* [47].

Previous work to determine the mean length of conversion tracts at *ry* has utilized models that take into account the selection for longer tract lengths [48,49]; we employed a similar strategy. We sequenced the region flanking the selected site of conversion (*ry^531^* or *ry^606^*) in 29 wild-type and 18 *rec* noncrossover gene conversion events and determined which nonselectable polymorphisms had also been converted (co-convertants: Figure 5B and C). To calculate the mean conversion tract length for each genotype, we derived a maximum likelihood model that incorporates standard errors, allowing us to compare the mean tract lengths of wild-type and *rec* mutants with 95% confidence intervals (see Materials and Methods). Conversion tract lengths generated by *rec* mutants are shorter than those from wild-type, with mean tract lengths of 250 basepairs (bp) and 441 bp, respectively. Although the 95% confidence intervals (160–340 bp and 323–558 bp, respectively) overlap slightly, the difference in tract lengths between *rec* and wild-type is statistically significant (*p* = 0.01). The increased recovery of noncrossover gene conversions from *rec* mutants therefore reflects an actual increase in the frequency of events and is not the result of longer conversion tracts. Indeed, because the conversion tracts are significantly shorter in *rec* mutants, the frequency of events recovered actually underestimates the true increase in noncrossover gene conversions.

Gene conversion tracts are the product of hDNA formation and repair. To determine whether the shorter tract length in *rec* mutants is due to a defect in the repair of hDNA, we used two different methods to determine whether PMS, a readout for hDNA repair, occurs in wild-type or *rec* mutants. Since the *ry* gene product is non–cell autonomous, any *ry^+^* recombinant could be mosaic, containing genetic information for both *ry^+^* and *ry* in different cells. To test for germ-line mosaicism, recombinant progeny were mated to *ry* flies; germ-line mosaics produce both *ry* and *ry^+^* progeny. To test for somatic mosaicism, PCR was performed using primers specific for the *ry^606^* and *ry^531^* alleles. Mosaicism was not detected in any of the 29 noncrossovers recovered from wild-type females or the 18 noncrossovers from *rec* mutants. Since mosaicism has been detected using both methods in noncrossover events from *mei-9* mutants (SJR and JS, unpublished data), we conclude that PMS does not occur at an appreciable frequency in the absence of REC.

## Discussion

Understanding how crossovers form is crucial to understanding the mechanisms eukaryotes use to faithfully pass half of their genetic information to the next generation. In *Drosophila,* many components of the meiotic recombination pathway have been identified, but a complete picture of the process has yet to emerge. In this paper, we molecularly and genetically characterized an important participant in this pathway—REC, the *Drosophila* homolog of MCM8—giving us new insight into requirements for crossover formation.

Our data support a model in which REC acts at an intermediate step of meiotic recombination. REC is not required for pre-meiotic S phase because homologous chromosomes in *rec* mutant females form normal synaptonemal complex, indicative of complete replication of genomic DNA [18]. Our finding that *rec* mutant females have about twice the normal number of noncrossover gene conversions indicates that initiation of recombination is not impaired in *rec* mutants; rather, very few DSBs are repaired as crossovers. Our data suggest that REC functions after strand invasion, since females mutant for both *rec* and *spn-A,* which encodes the Rad51 ortholog, phenocopy *spn-A* single mutants. Based on the distribution of residual crossovers in *rec* mutants and in *mei-9; rec* double mutants, it is likely that REC does not function with MEI-9 at resolution but acts at some previous step.

Normally, some recombination events become crossovers and some become noncrossovers. An increase in noncrossovers would occur if the crossover pathway were blocked so that most or all events followed the noncrossover pathway. In the *ry* intragenic recombination assay, noncrossover gene conversions are recovered only if they span a mutant site and convert that site to the wild-type sequence. In contrast, a crossover can be recovered if it occurs anywhere between the two mutations, as long as it generates a wild-type chromosome. Based on conversion tract lengths and the distance between the two mutations, we expect that many of the crossovers we recover would not be detected if they instead became noncrossovers, because they would not contain a conversion tract long enough to span a mutant site. The increase in noncrossovers that we observed in *rec* mutants, therefore, appears to be more than expected from this simple interpretation. A possible explanation for the increased frequency of noncrossovers in *rec* mutants comes from a hypothesis proposed by Bhagat et al. [41], who suggested that crossover distribution is disrupted as the result of a feedback mechanism that ensures one crossover per chromosome. The proposed feedback mechanism senses some intermediate in the crossover pathway (e.g*.*, the DHJ structure). In mutants in which this intermediate does not form, a signal causes the cell to initiate additional recombination events to ensure that a crossover is obtained. These initiations may occur outside the normal constraints, leading to a disruption of the normal distribution and an apparent polar reduction in crossing over. According to this model, *rec* mutants are impaired in formation of some crucial intermediate leading to crossovers. As a result, more recombination events are initiated, but most of these still become noncrossovers. Thus, the frequency of noncrossovers is elevated, and the crossovers that are produced do not follow the normal distribution.

The defect in *rec* mutants is not limited to an increased production of noncrossovers at the apparent expense of crossovers. We also found that noncrossover gene conversion tract length is significantly reduced in *rec* mutants. This could result from a defect in generating hDNA or a defect in repairing hDNA. Defects in repair of hDNA result in PMS of markers within the heteroduplex tract. We did not detect PMS in any of the events from wild-type or *rec* mutant females. Thus, *rec* mutants are not defective in repair of hDNA; rather, formation of hDNA may be compromised.

The length of hDNA can be affected by the extent of strand invasion and the amount of repair synthesis. In *S. cerevisiae,* the Mer3 helicase has been shown in vitro to stimulate Rad51-mediated strand invasion [50]. As in *rec* mutants, mutations in the gene that encodes Mer3 cause a reduction in the frequency of crossovers and an increase in the frequency of noncrossovers [51]. However, in physical assays *mer3* mutants are defective in the transition from DSB to strand invasion intermediate. Our data suggest that REC acts after strand invasion, so we do not favor the notion that REC performs a function similar to that of Mer3. Furthermore, based on the similarity of REC to replicative MCMs, we think it plausible that *rec* mutants have shorter conversion tracts because repair synthesis is diminished.

What is the relationship between reduced repair synthesis and decreased crossing over in *rec* mutants? In *S. cerevisiae,* crossovers are believed to arise through resolution of the DHJ intermediate. Although this process can also give rise to noncrossovers, most noncrossovers are thought to arise through SDSA [2]. There is evidence in *S. cerevisiae* that formation of a DHJ intermediate requires more repair synthesis than SDSA [3]. If this is also the case in *Drosophila,* then decreased repair synthesis would increase the probability that a meiotic DSB will be repaired through SDSA instead of the DHJ pathway.

**Figure 6:**
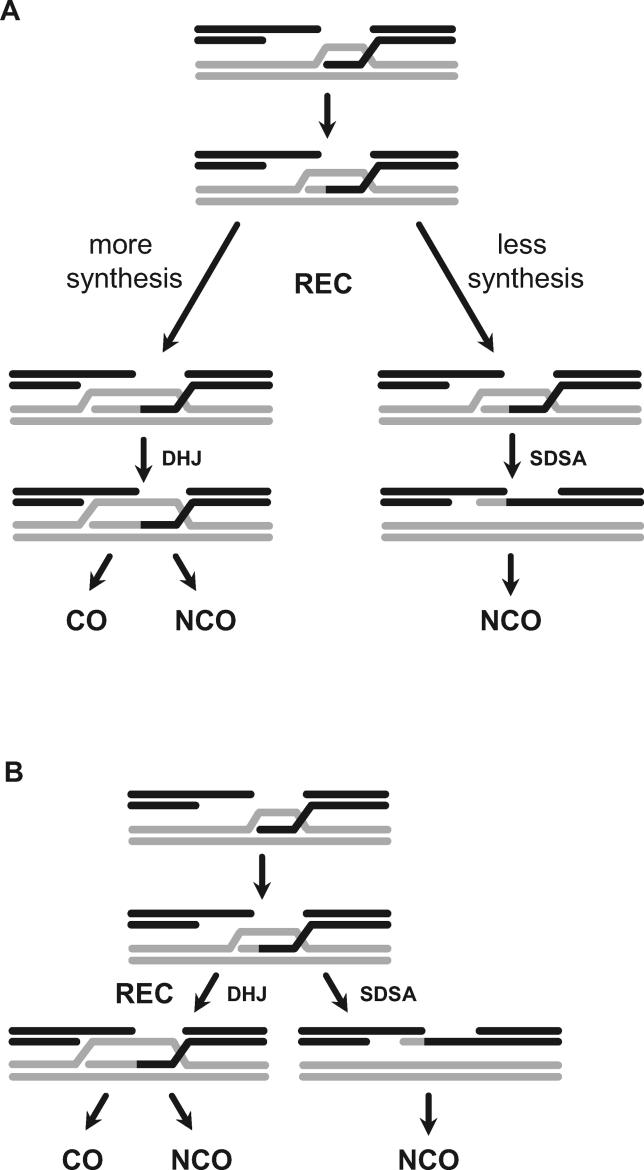
Models for REC Function in Meiotic Recombination (A) REC facilitates repair synthesis at all sites of meiotic recombination. Longer synthesis tracts allow second-end capture leading to DHJ intermediate formation, which can be resolved to generate COs and NCOs. If there is less repair synthesis, the newly synthesized strand can dissociate, anneal to the other broken end, and give rise to a NCO via SDSA. (B) REC is present only at sites that will mature into DHJ intermediates. After initial repair DNA synthesis, some single-end invasions dissociate and anneal to the broken chromosome giving rise to NCOs via SDSA. Other single-end invasion, intermediates produce more repair synthesis in a REC-dependent manner to give rise to DHJs that can be resolved to generate either a CO or a NCO.

We propose a model in which REC drives crossover formation by acting at the repair synthesis step of meiotic recombination (Figure 6). In the absence of REC, synthesis does not proceed far enough to allow second-end capture and formation of the DHJ intermediate, resulting in a deficit of crossovers. Noncrossovers may still be formed through SDSA. There are two versions of this model. First, REC may facilitate repair synthesis at all sites of recombination (Figure 6A). In this version, noncrossovers may normally arise through the DHJ pathway or the SDSA pathway, but in *rec* mutants the SDSA pathway is favored; the decrease in gene conversion tract length in *rec* mutants reflects an overall decrease in repair synthesis. Alternatively, REC may facilitate synthesis only at those recombination sites designated to become DHJ intermediates (Figure 6B). In this version of the model, sites lacking REC in wild-type flies undergo SDSA. The decrease in mean tract length in *rec* mutants is due to loss of those noncrossovers that would have arisen via a DHJ intermediate.

Our data do not indicate whether noncrossovers in wild-type flies arise through SDSA, DHJ, or a combination of the two. In *Drosophila,* SDSA is a primary pathway for DSB repair in nonmeiotic cells [16,17]. It may be that SDSA is the “default” pathway for recombinational repair of DSBs, and that meiosis-specific modifications promote formation of DHJs to allow crossing over. REC does not appear to play a role in SDSA in nonmeiotic cells (JS and M. Adams, personal communication), and therefore REC may be a component of the meiosis-specific modifications to DSB repair in *Drosophila*. To better understand the role of REC and the process of meiotic recombination, it will be important to determine the source of noncrossover recombinants in wild-type females.

## Materials and Methods

### Genetics.

Flies were propagated at 25 °C on standard medium containing agar, cornmeal, dextrose, and brewer's yeast. Information on loci mentioned, but not described here, is available on FlyBase (http://flybase.bio.indiana.edu/search/) [34].

### Screen for new *rec* alleles.

Two- to three-day-old *y/y^+^Y ; kar ry^506^ cv-c* males were starved for 1 h in an empty vial and transferred to a bottle containing 25 mM EMS in 1% sucrose. After eating EMS overnight, the males were transferred to a clean bottle for 1 d. Treated males were crossed to *y ; MKRS/TM6B Hu ry* females (60 females and 30 males per bottle). Single males with a balanced mutagenized Chromosome 3 were crossed to *y ; ru mus312 Z2035 st rec^1^ Ubx^bx34e^/TM6B Hu ry* females. Nonvirgin females that were y ; ru mus312 Z2035 st rec^1^ Ubx^bx34e^/* kar ry cv-c were placed in a new vial with brothers. The progeny were screened for non-yellow females and yellow males which result from X chromosome nondisjunction. Stocks were made of all lines that exhibited nondisjunction, and new alleles of *rec* were confirmed by complementation analysis. *Z2035* denotes an uncharacterized meiotic recombination mutation recovered in a screen of a collection of EMS-mutagenized chromosomes (SM and JS, unpublished data).

### Phylogenetic analysis of MCM domain proteins.

MCM proteins were identified by BLASTP and TBlastN searches of the genome sequence databases at http://www.ncbi.nlm.nih.gov/BLAST/. Accession numbers of each protein are listed in Table S1. Initial alignments were done with ClustalX [52], using the default settings. Regions outside of the core MCM domain were removed, as were large insertions within the core. A de novo alignment was generated with these core sequences, using the BLOSUM scoring matrices. This alignment was edited, and used to generate phylogenetic trees using the neighbor-joining method implemented in ClustalX, with 10,000 boot-strap trials, and independently using the maximum likelihood method of Tree Puzzle [53].

### Crossover frequency and distribution.

Crossing over on Chromosome 2 was assayed by crossing *al dp b pr Bl cn/+* females in different genetic backgrounds to *al dp b pr cn/CyO* males. Recombination frequencies are expressed in map units; 1 map unit is equal to a recombination frequency of 1%.

### Sequencing of *rec* mutants.

Single homozygous mutant flies were homogenized in Squishing Buffer (10 mM Tris-HCl [pH 8.2], 1 mM EDTA, 25 mM NaCl, 1 mg/ml Proteinase K) incubated at 37 °C for 30 min and then 95 °C for 5 min. PCR was performed using gene-specific primers. Products were isolated by agarose gel electrophoresis, purified from a gel slice, and sequenced at the University of North Carolina Genome Analysis facility. Mutations were verified by sequencing the opposite strand from a independent amplification.

### Intragenic recombination at the *rosy* locus.

Females *trans*-heterozygous for *ry^606^* and *ry^531^* were crossed to *y/y^+^Y ; kar ry^506^ cv-c* males (see Figure 4 for all crosses used). Crosses were set up in bottles containing 25 ml of food medium using 90 females and 30 males (*rec* mutants) or 30 females and 10 males (wild-type). After 3 d, the adults were transferred to new bottles to generate the second brood, and the first brood bottles were treated with 0.75 ml of 0.2% purine. This was repeated to generate a third brood. One out of every 25 bottles was not treated with purine so that the total number of flies screened could be estimated.

For every *ry^+^* progeny recovered, the type of recombinant was determined by examining the presence of flanking markers *kar* (0.3 map units to the right of *ry*) and *cv-c* (2.1 map units to the left of *ry*). Crossover progeny are wild-type for all three markers; a noncrossover gene conversion of *ry^531^* produces a crossveinless *(cv-c)* fly, and a noncrossover gene conversion of *ry^606^* results in a karmoysin *(kar)*-eyed fly. Because of the proximity of these markers to one another, it is unlikely that individual *ry^+^* progeny represent more than one recombination event in the region. Recombinant progeny were crossed to *kar ry^506^ cv-c* flies. After mating the recombinant fly, it was removed and homogenized in Squishing Buffer. To determine whether PMS occurred, allele-specific PCR was performed on the noncrossover gene conversion progeny using primers specific to either the *ry^531^* or *ry^606^* allele.

In all experiments, the cross to generate females *trans*-heterozygous for *ry^606^* and *ry^531^* was done at 25 °C. When adults began to emerge, bottles were kept at 18 °C overnight for virgin collection, and 25 °C during the day. For wild-type, crosses of these females to *ry^506^* males were incubated at room temperature, typically 20–22 °C. We analyzed 22 conversion tracts from these crosses. Crosses of *rec* mutant females to *ry^506^* males were incubated at 25 °C. We expected that the difference in temperatures would not have a strong effect on meiotic recombination, since recombination occurs about 4 d prior to mature oocyte formation, which in most cases would be during virgin collection. Nonetheless, to ensure that the decreased tract length in *rec* mutants was not due to the different temperatures, we generated eight noncrossover gene conversions from wild-type females at 25 °C. These conversion tracts were in fact longer than those from the room temperature experiments (mean of 666 bp versus 374 bp). Because of this small sample size, however, we have based our analysis on all 29 wild-type tracts. We note that if tracts are truly longer at 25 °C, then the decreased tract length in *rec* mutants is even more severe than our analysis suggests.

### Statistical analysis of gene conversion tract lengths.

We assume that the number of co-conversions follow a binomial distribution with parameter *φ^k^*, where *k* is the number of bp between the selected and nonselected sites. In this analysis, we consider 44 distinct values of *k*. The values of *k*, co-conversions, and total conversions for all the wild-type data are given in Table S3. Let *c_i_* be the number of co-conversions at the *ith* site, and let *n_i_* be the total number of conversions. The likelihood of the entire dataset is given by a product of binomial probabilities:


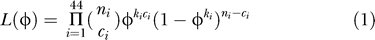


The log of the likelihood in (1) is given by


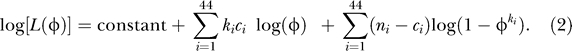


To find the maximum likelihood estimate of *φ*, we take the derivative of (2) with respect to *φ* and set it equal to zero. We denote the maximum likelihood estimate of *φ* by 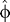 . Using standard large sample theory arguments [54], it can be shown that 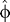 is approximately normally distributed with large sample variance approximately equal to


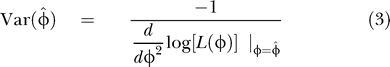


where


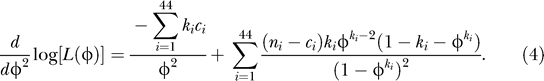


The formulae in (3) and (4) facilitate the computation of large sample confidence intervals for *φ*, which take the form 
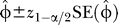
 , where 
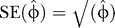
 and 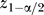 is the appropriate percentile of the standard normal distribution. For example, for a 95% confidence interval, α = 0.05 and *z_0.975_* = 1.96.

We follow Hilliker et al*.* [49] and assume that the tract length distribution follows a geometric distribution with parameter *φ*, and hence the mean tract length is 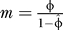 and the expected selected tract length is 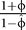 . Using standard large sample theory along with the delta method [54], it can be shown that 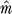 is approximately normally distributed with approximate large sample variance


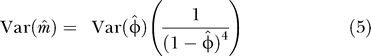


and thus the (1 – α) × 100% confidence interval for *m* is given by 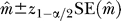 , where 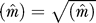 .

Using the above results, we can formally test for differences between the mean tract lengths between any two datasets *i* and *j* using a Z-statistic, which takes the form


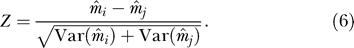


All computations of *φ* were done in XLIPSTAT [55] using double precision. The maximum likelihood estimate was computed in XLIPSTAT using the Nelder-Mead algorithm, which converged within 500 iterations for all datasets using a tolerance level of 10^−10^.

## Supporting Information

Figure S1 Alignment of the Most Highly Conserved Region of MCM Proteins This alignment shows the central region from all MCMs from *Homo sapiens (Has), Drosophila melanogaster (Dma), Arabidopsis thaliana (Ath), Entamoeba histolytica (Ehi),* and *Giardia lamblia (Gla);* the single MCM from the archaeal species *Sulpholobus sulfataricus (Ssu);* and MCM8 and MCM9 from *Anopheles gambiae (Aga), Drosophila pseudoobscura (Dps), Drosophila virilis,* and *Encephalitozoon cuniculi (Ecu).* A consensus is shown below the alignment, in which U, bulky aliphatic (I, L, M, V); @, aromatic (F, W, Y); &, bulky hydrophobic (I, L, M, V, F, W, Y); dot, any residue or no strong consensus. For this figure, a consensus residue is defined as one that is found in more than 80% of the sequences shown. Since 14 of the 44 sequences shown are from MCM8 and MCM9 orthologs, this figure emphasizes positions that are conserved between these more divergent subfamilies and canonical MCMs. Residues that match the consensus are shown in white text on a black background; conserved substitutions from the consensus are shown as white text on a gray background. The positions of the Walker A and B boxes and the arginine finger (RF) are indicated.
(43 KB PDF)

Figure S2 Phylogenetic Analysis of Eukaryotic MCM Family Proteins The tree shown was generated by the neighbor-joining method of ClustalW, using the alignment of the most highly conserved region of the MCM core domain shown in Figure S1 (correcting for multiple substitutions but including positions with gaps; unrooted). Note that *Giardia* MCM2 clusters with MCM8 and MCM9 in this analysis. The numbers on each node are the percentage of trees with the given branch from 10,000 independent boot-strapped iterations. The scale represents the relationship of branch length to phylogenetic distance expressed as the number of substitutions per site. See Figure S1 legend for species names.
(13 KB PDF)

Table S1 Sequences Used for Phylogenetic Analysis (40 KB DOC)

Table S2 Polymorphisms Used for Conversion Tract Length Determination (80 KB DOC)

Table S3 Co-Conversion Data Used for Conversion Tract Length Determination (67 KB DOC)

### Accession Numbers

The FlyBase (http://flybase.bio.indiana.edu/search/) accession numbers for genes and gene products discussed in this paper are *c(3)g* (FBgn0000246), ERCC1 (FBgn0028434), MEI-9 (FBgn0002707), MEI-218 (FBgn0002709), MEI-P22 (FBgn0016036), MEI-W68 (FBgn0002716), MUS-312 (FBgn0002909), *rosy(ry)* (FBgn0003308), *spn-A* (FBgn0003479), and *spn-E* (FBgn0003483).

## Abbreviations

bp: basepair
DHJ: double Holliday junction
DSB: double-strand break
EMS: ethyl methanesulfonate
hDNA: heteroduplex DNA
MCM: mini-chromosome maintenance
PMS: post-meiotic segregation
SDSA: synthesis-dependent strand annealing
