# Supplementary material for: REC, Drosophila MCM8, Drives Formation of Meiotic Crossovers
Source: PLoS Genet. 2005 Sep 23;1(3):e40. doi: 10.1371/journal.pgen.0010040 (PMC1231718; doi:10.1371/journal.pgen.0010040)
Supplement: Supplementary file 1 [file pgen.0010040.sg001.pdf]

```
                                         Walker A
Dme REC : KAACLLSLLGGKGAET---------EAINVLLVGDPGIGKTKILQSCAQIAERGAHVSGKRGAQSAQQLGVTFAGRNKRV-LQAGSLMMA
Dps REC : KAACLLSLLGGGA------------GGVNVLLVGDPGIGKSKILQNCAQITERGSHISGKRGAQSAQRLGPTFTGRNKRV-LQAGGLMTA
Dvi REC : KAACLLSLLGE---------------NINVLLVGDPGIGKSMMLQNCAQITERGAHVSGKRGAQAANQLGISFCGRNKRV-MDAGALLMA

Aga MCM8: KAGLLLGLFGGMANTAR------TRAEIHVLVVGDPGIGKSQILQSCANVSPRGIFVCGTNSSNVGLTVTVRTEKGVG-ASLEAGALVLA
Hsa MCM8: KAGLALALFGGSQKYADDKNRIPIRGDPHILVVGDPGLGKSQMLQAACNVAPRGVYVCGNTTTTSGLTVTLSKDSSSGDFALEAGALVLG
Ath MCM8: KAGITLSLFGGVRKHSMDRNKVPVRGDIHVLIVGDPGLGKSQLLQAAAAISPRGIYVCGNATTRAGLTVAVVKDSMTNDYAFEAGAMVLA
Ecu MCM8: KVGLVLSLFGG---TKKSAGQHSIRSETHVLIVGDPGLGKSRLLLSTCGILPKSSYVSGSFTTTAGLTVSLTHDPVSGEYMADAGALVVA
Ehi MCM8: KTAIVLVLFGG----TRKHDIAKIRADSHLLIVGDPGLGKSQMLRAVANIVPRGVYVSGSSTTKTGLTVALHRYSGTSDFTLESGALVLG

Hsa MCM9: KLAVAMVLAGGIQRTDATGT--RVRGESHLLLVGDPGTGKSQFLKYAAKITPRSVLTTGIGSTSAGIVCDNFK
Ath MCM9: KLAVALTLIGGVQHVDAS--------ESHLLLIGDPGTGKSQFLKFAAKLSNRAVITTGLGSTSAGLTVTAVKD--GGEWMLEAGALVLA
Aga MCM9: KLAIALALASCTERVGDGEQQATVRGHSHLLLVGDPGLAKSQLLKYASEIASRAVYTTGMGCSSAGLTAAAVKD--EGEWQLEAGALVLA
Ecu MCM9: KLGLLLALVSG---SHGADQKSGTRSNSHILLVGDSGTGKSHLLKTCSRLLSPAVLTNGVGTTQAGLTTCAVRQG--REWVLEAGALVLA
Ehi MCM9: KLAVMLVATGGISRIDKES-------TIHLLLVGDPGTGKSQLLKFASKLGPRHVQTTGGGTTSAGLTVSVINI--GGELSLDAGALVLA

Hsa MCM5: KKAIACLLFGGSRKRLPDG--LTRRGDINLLMLGDPGTAKSQLLKFVEKCSPIGVYTSGKGSSAAGLTASVMRDPSSRNFIMEAGAMVLA
Dme MCM5: KKAITCMLFGGSRKRLPDG--LCRRGDINVLLLGDPGTAKSQLLKFVEKVAPIAVYTSGKGSSAAGLTASVMKDPQTRNFVMEAGAMVLA
Ath MCM5: KRAAACLLFGGSRKSLPDG--VKLRGDINVLLLGDPSTAKSQFLKFVEKTAPIAVYTSGKGSSAAGLTASVIRDSSTREFYLEGGAMVLA
Ehi MCM5: KEAVLCLMLGGSGKALPDG--THLRGDINVLLMGDPGTAKSQLLKFVQMATPIGVYTSGKGSSAAGLTAAVNKDSSTGEFYLEGGALVLG
Gla MCM5: KRAIACLLFGGTNKYTQE----LLRGNINVLLISDPGLGKSELLLEASRLAPIGIYTSGKSTSAVGLTAGVMRDKATSEFFLEGGALVLA
Hsa MCM2: KRGLALALFGGEPKNPGGK--HKVRGDINVLLCGDPGTAKSQFLKYIEKVSSRAIFTTGQGASAVGLTAYVQRHPVSREWTLEAGALVLA
Dme MCM2: KRALALALFGGESKNPGEK--HKVRGDINLLICGDPGTAKSQFLKYTEKVAPRAVFTTGQGASAVGLTAYVRRNPVSREWTLEAGALVLA
Ath MCM2: KTALALAMFGGQEKNIKGK--HRLRGDINVLLLGDPGTAKSQFLKYVEKTGQRAVYTTGKGASAVGLTAAVHKDPVTREWTLEGGALVLA
Ehi MCM2: KAAIALALFGGEQRVLVDKGNHRTRGDINVLLLGDPGTAKSQLLKYSQKLAPRAVFTTGRGSTAVGLTAAVKKDSMNGEWALEGGALVLA
Gla MCM2: KQAILMALVGGISALDGGAASRFTRGDLHMLILGDPGVSKSQLLKYVQHISPKCVYTSGKGSSAAGLTVSVKKSSVTGEFYLQAGALVLA
Hsa MCM3: KKAILCLLLGGVERDLENG--SHIRGDINILLIGDPSVAKSQLLRYVLCTAPRAIPTTGRGSSGVGLTAAVTTDQETGERRLEAGAMVLA
Dme MCM3: KQAILCLLLGGVEKILPNG--TRLRGDINVLLIGDPSVAKSQLLRYVLNTAPRAIPTTGRGSSGVGLTAAVTTDQETGERRLEAGAMVLA
Ath MCM3: KKAVVLLMLGGVEKNLKNG--THLRGDINMMMVGDPSVAKSQLLRAIMNIAPLAISTTGRGSSGVGLTAAVTSDQETGERRLEAGAMVLA
Ehi MCM3: KKAILLMLVGATPKIRLR---SRVRGDIHVMLCGDPSTAKSQLLRYVMSIAPLAVSTNGRGATGVGLTAAVVNDPDTNQRTLEAGAMVLA
Gla MCM3: KRGIVLQLLQGVPREFSSS--SRIRGDINILLIGDPGSAKSQMLRVVKQLMPVSVQTTGRGSSGVGLTAAVVIDGTTGERRLDPGAAVLA
Hsa MCM7: KKALLLLLVGGVD-QSPRG--MKIRGNINICLMGDPGVAKSQLLSYIDRLAPRSQYTTGRGSSGVGLTAAVLRDSVSGELTLEGGALVLA
Dme MCM7: KKALLLLLVGGVD-KRPDG--MKIRGNINICLMGDPGVAKSQLLGYISRLAVRSQYTTGRGSSGVGLTAAVMKDPLTGEMTLEGGALVLA
Ath MCM7: KKALLLLLVGAPHRQLKDG--MKIRGDVHICLMGDPGVAKSQLLKHIINVAPRGVYTTGKGSSGVGLTAAVMRDQVTNEMVLEGGALVLA
Ehi MCM7: KKALLLTVVGAPTRRMKDG--VSIRGDINTLLVGEPGIAKSQLLRAVAGVAPRSVYTTGKGSSGAGLTAAVIRDQLTKEWVLEGGALVLA
Gla MCM7: KLACLCSLVGGNEIVVPDM---KVRGNINTLFVGDPGCAKSALLKFTCTIAERGIYVAGRGASGAGLTTAAIRIPGTTDYSLEGGALVIA
Hsa MCM6: KRGVLLMLFGGPKTTG-EG--TSLRGDINVCIVGDPSTAKSQFLKHVEEFSPRAVYTSGKASSAAGLTAAVVRDEESHEFVIEGGALMLA
Dme MCM6: KRGILLQQFGGAKTTT-EK--TSLRGDINVCIVGDPSTAKSQFLKQVSDFSPRAIYTSGKASSAAGLTAAVVRDEESFDFVIEAGALMLA
Ath MCM6: KRAVLLMLLGGHKTTH-EG--INLRGDINVCIVGDPSCAKSQFLKYTAGIVPRSVYTSGKSSSAAGLTATVAKEPETGEFCIEAGALMLA
Ehi MCM6: KKGILLLLFGGHKTTK-EG--IALRGDINICVIGDPSTAKSQFLKCVSTIHPRCIYTSGKASSAAGLTAAVLKDPETGDFNIEAGAMMLA
Gla MCM6: KLGILLQLLGGKKITRSEH--LSIRSDINILLIGDPSTAKSQLLQYTADFHQKAVYTSGKSSTAAGLTAAVVTDPDTGEYTVEAGALIRA
Hsa MCM4: KKGILLQLFGGTRKDFSH---TKFRAEINILLCGDPGTSKSQLLQYVYNLVPRGQYTSGKGSSAVGLTAYVMKDPETRQLVLQTGALVLS
Dme MCM4: KKGILLQLFGGTKKKHAT---LNFRSEIHLLLCGDPGTSKSQMLQYVFNLVPRSQYTSGRGSSAVGLTAYVTKDPETRQLVLQTGALVLA
Ath MCM4: KKGLLCQLFGGNALNLAS---GNFRGDINILLVGDPGTSKSQLLQYIHKLSPRGIYTSGRGSSAVGLTAYVAKDPETGETVLESGALVLS
Ehi MCM4: KKGLLCQLFGGSAKTLQD---NKLRGDINILMLGDPGTSKSQLLTFMHKVAPRGMYTSGRGSSAVGLTAFVGKS-EEGGTVLESGALVMS
Gla MCM4: KTGLLLQCFGGVQKDS-------YRGQIHVLLVGDPGLAKSKLLQYVAKISPRSVYTSGKGSSQAGLTATVSRHPETHEFYLDPGALLLS
Ssu MCM : KEALALALFGGVPKVLED---TRIRGDIHILIIGDPGTAKSQMLQFISRVAPRAVYTTGKGSTAAGLTAAVVREKGTGEYYLEAGALVLA

consensus K.AU...U&GG.............R..INUUUUGDPG.AKSQ&L............TSG...S..GLT..V..........UEAGAUUUA
            G                         H       S G             VT   T                    DG
```

```
              Walker B                                                                                RF
Dme REC : SGGGHCTLDDVDKLASKQAV-LLQCLQSEEVNLPLAGAFASFPAQPSVIACANPQRGQYDE---GRYLLQNINISPSLLREFHLVYILLD
Dps REC : SSPGHCTLDDVDKLASKQAV-LLQCMQSGEINMPLPGAFSTFLAQPSIIACANPQRGQYDE---GRYLLQNIHIVPSLLKEFHLVYVLLD
Dvi REC : GTAGTCLVDGVDKLASKQTL-LLQCMQAGELNMPLPGLFASFEAQPAIIACANPQRGQYDQ---ARYLLQNIRITAALLKEFHLVYVLLD

Aga MCM8: DQGV-CCIDEFDKMS--GHSGLLEVMEQRSVSVAKAGVICTVPARTTVLAAANPAGGHYNK---AKTVSENLKLHPALLSRFDLVFILLD
Hsa MCM8: DQGI-CGIDEFDKMGN-QHQALLEAMEQQSISLAKAGVVCSLPARTSIIAAANPVGGHYNK---AKTVSENLKMGSALLSRFDLVFILLD
Ath MCM8: DGGL-CCIDEFDKMTT-EHQALLEAMEQQCVSVAKAGLVASLSARTSVIAAANPVGGHYNR---AKTVNENLKMSAALLSRFDLVFILLD
Ecu MCM8: DNGI-CCLDEFDKID--DHAALFEAMEDQKVSIAKGGVICSVPTRATVIAATNPRHGHFDR---GKTMAENIRFDPGLLSRFDLIFLLLD
Ehi MCM8: DQGV-CCIDEFDKMERADYSSLLEAMEQQSISIAKAGICCTLLARTSVIAAANPVEGHFNC---GKTVSENINMPSPLLSRFDLIFVLVD

Ath MCM9: DGGL-CCIDEFDSMREHDRATIHEAMEQQSISVAKAGLVTTLSTKTIVFGATNPK-GQYDPDQYTKNPEWDAVLSVQIEQDREVDDLTTI
Aga MCM9: DGGI-CCIDEFNLMRETDKASIHEAMEQQTISVAKAGMVCKLSTRCVVLAATNPK-NLYTMSDGLGKSAENIGIGGPLLSRFDMVMILKD
Ecu MCM9: DTGL-CCIDEFNKLKVNEKNGLLEAMEQQTLSIAKAGIVSSLNTRCSVIAAINTRHKYSFN---KSISENIIVATP-LISRFDLIFGLFD
Ehi MCM9: DGGV-CCIDEFSGINKSDRADIHEAMEQQTLSVAKAGIVSQLHTRTAILAATNPK-GRYDP---TKSMSLNTAIDPPLLSRFDIILLLLD

Hsa MCM5: DGGV-VCIDEFDKMREDDRVAIHEAMEQQTISIAKAGITTTLNSRCSVLAAANSVFGRWDE---TK-GEDNIDFMPTILSRFDMIFIVKD
Dme MCM5: DGGV-VCIDEFDKMREDDRVAIHEAMEQQTISIAKAGITTTLNSRCSVLAAANSIFGRWDD---TK-GEENIDFMPTILSRFDMIFIVKD
Ath MCM5: DGGV-VCIDEFDKMRPEDRVAIHEAMEQQTISIAKAGITTVLNSRTSVLAAANPPSGRYDD---LKTAQDNIDLQTTILSRFDLIFIVKD
Ehi MCM5: DGGV-VCIDEFDKMNEVDRVAIHEAMEQQTISIAKAGITAVLNARSAVLAAANPSFGRFNE---RASFGDNVNLKTTILSRFDMIFMIRD
Gla MCM5: DKGI-VCIDELDKMNETDRVALHEAMEQGSISISKAGISTTLNARTSILAAANPTLGRFDD---FQKAADQIDFSVTILSRFDLVFMLKD
Hsa MCM2: DRGV-CLIDEFDKMNDQDRTSIHEAMEQQSISISKAGIVTSLQARCTVIAAANPIGGRYDP---SLTFSENVDLTEPIISRFDILCVVRD
Dme MCM2: DQGV-CLIDEFDKMNDQDRTSIHEAMEQQSISISKAGIVTSLQARCTVIAAANPIGGRYDP---SMTFSENVNLSEPILSRFDVLCVVKD
Ath MCM2: DRGI-CLIDEFDKMNDQDRVSIHEAMEQQSISISKAGIVTSLQARCSVIAAANPVGGRYDS---SKSFAQNVELTDPILSRFDILCVVKD
Ehi MCM2: DEGV-CLIDEFDKMDDQDRTSIHEAMEQQSISISKAGIVTSLKARCSVIAAANPKTGKYNP---NKNLNQNVNLTEPIISRFDLIMIVRD
Gla MCM2: NGGI-CIIDELDKMNEIDRTALHQAMEQQTVSVAKAGIISTLEARAGIIAAANPVSGQYVS---SLPVTCNLNIGDALMSRFDLICVVKD
Hsa MCM3: DRGV-VCIDEFDKMSDMDRTAIHEVMEQGRVTIAKAGIHARLNARCSVLAAANPVYGRYDQ---YKTPMENIGLQDSLLSRFDLLFIMLD
Dme MCM3: DRGV-VCIDEFDKMSDIDRTAIHEVMEQGRVTISKAGIHASLNARCSVLAAANPVYGRYDQ---YKTPMENIGLQDSLLSRFDLIFVMLD
Ath MCM3: DKGI-VCIDEFDKMNDQDRVAIHEVMEQQTVTIAKAGIHASLNARCSVVAAANPIYGTYDR---SLTPTKNIGLPDSLLSRFDLIFIVLD
Ehi MCM3: DRGI-VCVDEFDKMSIEDRAAMHEVMEQQTVTVQKAGIHTALNARCSILAAANPSNGNYDV---KKSPMENLYFPESLLSRFDLIFIILD
Gla MCM3: DKGV-LLIDEFDKVDADDRALLHEALEQQSISISKAGLHCTLNARCSVLAAANPVYGFFDP---KRSFAENIALPDSLLSRYDLVFLVRD
Hsa MCM7: DQGV-CCIDEFDKMAEADRTAIHEVMEQQTISIAKAGILTTLNARCSILAAANPAYGRYNP---RRSLEQNIQLPAALLSRFDLLWLIQD
Dme MCM7: DQGV-CCIDEFDKMADQDRTAIHEVMEQQTISIAKAGIMTTLNARVSILAAANPAFGRYNP---RRTVEQNIQLPAALLSRFDLLWLIQD
Ath MCM7: DMGI-CAIDEFDKMDESDRTAIHEVMEQQTVSIAKAGITTSLNARTAVLAAANPAWGRYDL---RRTPAENINLPPALLSRFDLLWLILD
Ehi MCM7: DMGI-CCIDEFDKMDETDRTAIYEVMEQQSISIAKAGITTSLNARVSIVAAANPIKARYDI---RKSVSENINLPAALVSRFDLLFVLLD
Gla MCM7: DQGV-CALDELDKLEEADRTAIYEVMEQGTISVAKAGITATLNARATVVAAANPKFSIWDP---SISVASNINIPEALISRFDILFVIRD
Hsa MCM6: DNGV-CCIDEFDKMDVRDQVAIHEAMEQQTISITKAGVKATLNARTSILAAANPISGHYDR---SKSLKQNINLSAPIMSRFDLFFILVD
Dme MCM6: DNGI-CCIDEFDKMDQRDQVAIHEAMEQQTISIARAGVRATLNARTSILAAANPINGRYDR---SKSLQQNIQLSAPIMSRFDLFFILVD
Ath MCM6: DNGI-CCIDEFDKMDIKDQVAIHEAMEQQTISITKAGIQATLNARTSILAAANPVGGRYDK---SKPLKYNVNLPPAILSRFDLVYVMID
Ehi MCM6: DNGV-CCIDEFDKMDYFNQVALHEAMEQQTISIAKGGLHATLNARAAVLAAANPLKGRYDS---NRSLKSNLNIGDALMSRFDLFFVVLD
Gla MCM6: DGGL-CLIDEFEKISVTDQTALHECLEQQSVSINKAGISITLKAKTPVLAAMNPIGSRYQR---NKSLKNNINISQPILSRFDLAFVLLD
Hsa MCM4: DNGI-CCIDEFDKMNESTRSVLHEVMEQQTLSIAKAGIICQLNARTSVLAAANPIESQWNP---KKTTIENIQLPHTLLSRFDLIFLLLD
Dme MCM4: DNGV-CCIDEFDKMNDSTRSVLHEVMEQQTLSIAKAGIICQLNARTSILAAANPAESQWNK---RKNIIDNVQLPHTLLSRFDLIFLVLD
Ath MCM4: DRGI-CCIDEFDKMSDSARSMLHEVMEQQTVSIAKAGIIASLNARTSVLACANPSGSRYNP---RLSVIENIHLPPTLLSRFDLIYLILD
Ehi MCM4: DKGL-CCIDEFDKMTEMTRSVLHEAMEQQTISVAKSGIVCSLNARTAILASANPKESRYNP---KLSVLDNIQMPPSLLSRFDLIYLILD
Gla MCM4: DGGI-CCLDEFDKSSEDVRSSLHEVMEHGQLSIAKAGILATLSAKTSILAAANPIDSCYNP---KRTVVQNLNLPPSLLSRFDLIYLLLD
Ssu MCM : DGGI-AVIDEIDKMRDEDRVAIHEAMEQQTVSIAKAGIVAKLNARAAVIAAGNPKFGRYIS---ERPVSDNINLPPTILSRFDLIFILKD

consensus D.GU.C.IDEFDKM.......U.E.MEQ..USU.KAGU...L..K..UUAAANP....@D..........NU.U...UUSRFDLU.UU.D
              V                                                    N
```
