# Supplementary material for: REC, Drosophila MCM8, Drives Formation of Meiotic Crossovers
Source: PLoS Genet. 2005 Sep 23;1(3):e40. doi: 10.1371/journal.pgen.0010040 (PMC1231718; doi:10.1371/journal.pgen.0010040)
Supplement: Supplementary file 2 [file pgen.0010040.sg002.pdf]

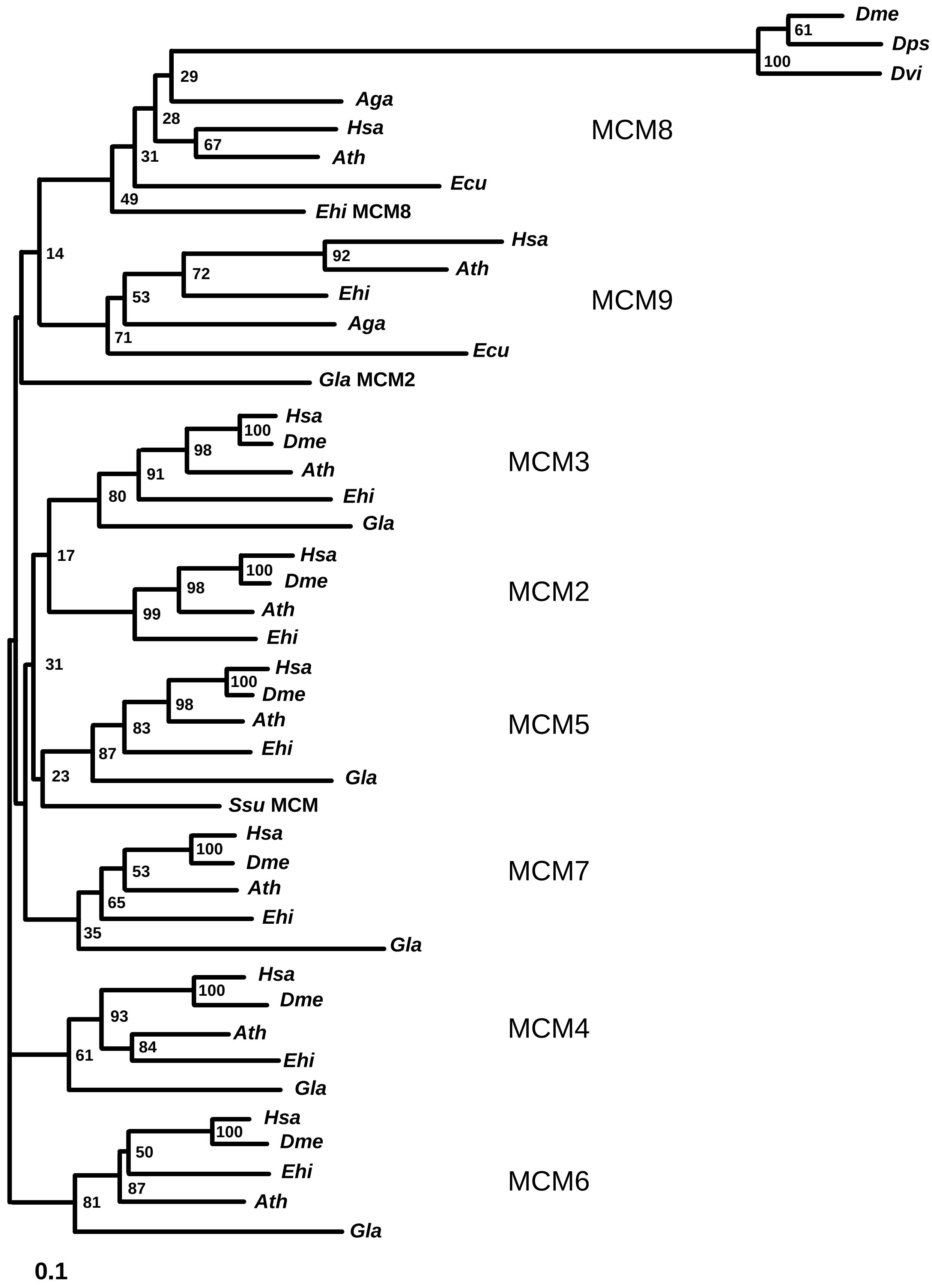
Dme
Dps
Dvi
61
100
29
Aga
28
Hsa
67
Ath
MCM8
31
Ecu
49
Ehi MCM8
Hsa
92
Ath
14
72
Ehi
53
MCM9
Aga
71
Ecu
Gla MCM2
Hsa
100
Dme
98
Ath
91
MCM3
80
Ehi
Gla
Hsa
17
100
Dme
98
MCM2
Ath
99
Ehi
31
Hsa
100
Dme
98
Ath
83
MCM5
Ehi
87
23
Gla
Ssu MCM
Hsa
100
Dme
53
Ath
MCM7
65
Ehi
35
Gla
Hsa
100
Dme
93
Ath
84
MCM4
61
Ehi
Gla
Hsa
100
Dme
50
Ehi
87
MCM6
Ath
81
Gla
0.1
