# Supplementary material for: REC, Drosophila MCM8, Drives Formation of Meiotic Crossovers
Source: PLoS Genet. 2005 Sep 23;1(3):e40. doi: 10.1371/journal.pgen.0010040 (PMC1231718; doi:10.1371/journal.pgen.0010040)
Supplement: Supplementary file 3 [file pgen.0010040.st001.doc]

**Table S1.**  Sequences Used for Phylogenetic Analysis

| **Species ^a^** | **MCM2 ^b^** | **MCM3** | **MCM4** | **MCM5** | **MCM6** | **MCM7** | **MCM8** | **MCM9** |
| --- | --- | --- | --- | --- | --- | --- | --- | --- |
| ***Hse*** | NP_004517.2  294-802 | NP_002379.2  113-653 | NP_005905.2  271-768 | NP_006730.2  137-649 | NP_005906.2  123-656 | NP_005907.3  149-641 | NP_115874.3  207-748 | NP_694987.1  112-391 |
| ***Dme*** | NP_477121.1  279-779 | NP_511048.2  110-647 | NP_477185.1  274-771 | NP_524308.2  132-648 | NP_511065.1  117-648 | NP_523984.1  149-641 | NP_732072.1  246-792 | NA |
| ***Cel*** | NP_496558.1  270-776 | NP_506706.1  118-650 | NP_490962.1  234-729 | NP_497858.1  137-672 | NP_499170.1  122-659 | NP_504199.1  161-651 | NA | NA |
| ***Sce*** | NP_009530.1  306-845 | NP_010882.1  168-737 | NP_015344.1  314-833 | NP_013376.1  148-688 | NP_011314.2  276-835 | NP_009761.1  227-724 | NA | NA |
| ***Ecu*** | NP_584768.1  202-687 | NP_597158.1  95-578 | NP_584640.1  143-621 | NP_585790.1  116-614 | NP_597420.1  112-623 | NP_597059.1  147-618 | NP_586386.1  108-607 | NP_585977.1  50-525 |
| ***Ath*** | NP_175112.1  312-819 | NP_199440.1  104-634 | NP_179236.3  346-748 | NP_178812.1  134-643 | NP_680393.1  120-655 | NP_192115.1  143-637 | NP_187577.1  145-677 | NP_179021.2  120-569 |
| ***Ehi*** | EAL50675.1  355-860 | EAL47986.1  10-530 | EAL43702.1  95-576 | EAL43398.1  134-621 | EAL48722.1  132-654 | EAL45421.1  160-654 | EAL48818.1  119-642 | EAL49553.1  12-503 |
| ***Pfa*** | NP_702065.1  306-844 | NP_703613.1  196-785 | NP_705078.1  294-908 | NP_701481.1  165-677 | NP_705435.1  210-750 | NP_704017.1  209-717 | NP_701477.1  172-927 | NP_702813.1  430-1389 |
| ***Lma* ^c^** | LmjF28.0850  315-861 | LmjF33.2700  136-692 | CAB59865.1  251-879 | LmjF24.0910  139-718 | LmjF28.2385  157-701 | CAB55370.1  144-645 | CAB89596.2  192-863 | LmjF35.4910  119-621 |
| ***Gla*** | EAA40971.1  343-967 | EAA36979.1  100-802 | EAA40854.1  185-715 | EAA38067.1  124-651 | EAA40537.1  139-792 | EAA40792.1  118-625 | NA | NA |

^a^ For abbreviations, see Figure 2 legend.

^b^ Accession and version numbers are given, along with coordinates of residues used in alignments. NA = not applicable

^c^ BLAST searches for *Leishmania major* were performed at the GeneDB site, http://www.genedb.org/genedb/leish/blast.jsp.
