# Supplementary material for: REC, Drosophila MCM8, Drives Formation of Meiotic Crossovers
Source: PLoS Genet. 2005 Sep 23;1(3):e40. doi: 10.1371/journal.pgen.0010040 (PMC1231718; doi:10.1371/journal.pgen.0010040)
Supplement: Supplementary file 4 [file pgen.0010040.st002.doc]

**Table S2.** Polymorphisms Used for Conversion Tract Length Determination

| **Position^a^** | **Genome^b^** | ***ry^531^*** | ***ry^606^*** |
| --- | --- | --- | --- |
| -3009 (**-3020***) | A | **G** | **A** |
| -2882 **(-2893*)** | A | **G** | **A** |
| -2871 **(-2882*)** | G | **T** | **G** |
| -2646 **(-2657*)** | T | **C** | **T** |
| -2565 ^ -2564 | - | ATAGCTTGGAG | ATAGCTTGGAG |
| -2562 | A | T | T |
| -2539 | A | G | G |
| -2399 | C | A | A |
| -2099 | A | G | G |
| -2069 | T | A | A |
| -**2010*** | C | **G** | **C** |
| -1891 | C | A | A |
| -1830 | C | T | T |
| -1827 | C | T | T |
| -1708 | C | T | T |
| **-1316*** | C | **C** | **T** |
| **-1061*** | A | **A** | **T** |
| **-1030 ^ -1029*** | - | **T** | **-** |
| **-996*** | T | **G** | **T** |
| **-937*** | A | **A** | **T** |
| -913 | G | A | A |
| **-910*** | G | **G** | **A** |
| **-698*** | T | **A** | **T** |
| **-686 ^ -685*** | ---- | **GGGT** | **----** |
| **-679*** | G | **A** | **G** |
| -677 | T | C | C |
| **-668*** | T | **A** | **T** |
| **-667*** | A | **G** | **A** |
| **-636*** | C | **A** | **C** |
| **-468*** *ry^606^* | G | **G** | **A** |
| **-44*** | C | **T** | **C** |
| **467*** | T | **C** | **T** |
| 689 | G | A | A |
| **736*** | G | **A** | **G** |
| **767*** | G | **A** | **G** |
| **777*** | G | **A** | **G** |
| 1032 | T | C | C |
| **1153*** | C | **C** | **T** |
| 1534 | A | T | T |
| 1559 | T | C | C |
| **1596*** | G | **G** | **T** |
| 1680 | G | C | C |
| **2653*** | G | **T** | **G** |
| 2710 | T | C | C |
| **2957*** | T | **T** | **C** |
| **3148*** | C | **C** | **T** |
| **3312*** *ry^531^* | G | **A** | **G** |
| 3388 | T | C | C |
| **3529*** | T | **T** | **C** |
| **3557*** | G | **G** | **A** |
| 3671 | T | G | G |
| **3703*** | T | **T** | **A** |
| **3723*** | C | **C** | **A** |
| 4008 | A | C | C |
| 4010 | G | T | T |
| 4011, 4012 | CA | -- | -- |
| 4032 (4030) | T | C | C |
| 4132 (4130) | A | C | C |
| 4222 (4220) | C | A | A |
| 4272 (4270) | A | T | T |
| 4417 (**4415***) | A | **A** | **C** |
| 4741 (**4739***) | A | **C** | **A** |
| 4779 (**4777***) | C | **G** | **C** |

^a^ Position of *ry* polymorphisms in the *Drosophila* genome sequence Release 4.1 (Genbank accession number AE003698). Positions in parentheses reflect the numbering of nucleotides in the *ry^531^* and *ry^606^* alleles; positions analyzed in this study are bold and starred. ^ represents the insertion of sequence. Position numbers are relative to an *Eco*RI site in the coding region of *ry*, using the coordinates in Bender et al. [52].

^b^ Sequence differences between the *Drosophila* genome sequence of *ry* and the alleles used in this study.
