# Supplementary material for: REC, Drosophila MCM8, Drives Formation of Meiotic Crossovers
Source: PLoS Genet. 2005 Sep 23;1(3):e40. doi: 10.1371/journal.pgen.0010040 (PMC1231718; doi:10.1371/journal.pgen.0010040)
Supplement: Supplementary file 5 [file pgen.0010040.st003.doc]

**Table S3.** Co-Conversion Data Used for Conversion Tract Length Determination

| **allele^a^** | **site^b^** | **bp^c^** | **co-conversions / total conversions^d^** | | |
| --- | --- | --- | --- | --- | --- |
|  |  |  | **w.t., RT** | **w.t., 25° C** | ***rec*** |
| 531 | 3148 | 164 | 6 /10 | 2 / 2 | 4 / 9 |
| 606 | -636 | 168 | 9 /12 | 5 / 5 | 2 / 9 |
| 606 | -667 | 199 | 8 /12 | 5 / 5 | 2 / 9 |
| 606 | -668 | 200 | 8 /12 | 5 / 5 | 2 / 9 |
| 606 | -679 | 211 | 8 /12 | 5 / 5 | 2 / 9 |
| 531 | 3529 | 217 | 5 /10 | 0 / 2 | 2 / 9 |
| 606 | -685 | 217 | 8 /12 | 5 / 5 | 2 / 9 |
| 606 | -698 | 230 | 8 /12 | 5 / 5 | 2 / 9 |
| 531 | 3557 | 245 | 5 /10 | 0 / 2 | 2 / 9 |
| 531 | 2957 | 355 | 2 /10 | 2 / 2 | 4 / 9 |
| 531 | 3703 | 391 | 4 /10 | 0 / 2 | 0 / 9 |
| 531 | 3723 | 411 | 3 /10 | 0 / 2 | 0 / 9 |
| 606 | -44 | 424 | 2 /12 | 2 / 5 | 2 / 9 |
| 606 | -910 | 442 | 3 /12 | 4 / 5 | 2 / 9 |
| 606 | -937 | 469 | 3 /12 | 4 / 5 | 2 / 9 |
| 606 | -996 | 528 | 2 /12 | 4 / 5 | 2 / 9 |
| 606 | -1029 | 561 | 1 /12 | 3 / 5 | 2 / 9 |
| 606 | -1061 | 593 | 1 /12 | 3 / 5 | 2 / 9 |
| 531 | 2653 | 659 | 0 /10 | 0 / 2 | 0 / 9 |
| 606 | -1316 | 848 | 1 /12 | 2 / 5 | 2 / 9 |
| 606 | 467 | 935 | 1 /12 | 2 / 5 | 0 / 9 |
| 531 | 4415 | 1103 | 1 /10 | 0 / 2 | 0 / 9 |
| 606 | 736 | 1204 | 1 /12 | 0 / 5 | 0 / 9 |
| 606 | 767 | 1235 | 1 /12 | 0 / 5 | 0 / 9 |
| 606 | 777 | 1245 | 1 /12 | 0 / 5 | 0 / 9 |
| 531 | 4739 | 1427 | 0 /10 | 0 / 2 | 0 / 9 |
| 531 | 4777 | 1465 | 0 /10 | 0 / 2 | 0 / 9 |
| 606 | -2010 | 1542 | 0 /12 | 0 / 5 | 1 / 9 |
| 606 | 1153 | 1621 | 1 /12 | 0 / 5 | 0 / 9 |
| 531 | 1596 | 1716 | 0 /10 | 0 / 2 | 0 / 9 |
| 606 | 1596 | 2064 | 1 /12 | 0 / 5 | 0 / 9 |
| 531 | 1153 | 2159 | 0 /10 | 0 / 2 | 0 / 9 |
| 606 | -2657 | 2189 | 0 /12 | 0 / 5 | 1 / 9 |
| 606 | -2882 | 2414 | 0 /12 | 0 / 5 | 0 / 9 |
| 606 | -2893 | 2425 | 0 /12 | 0 / 5 | 0 / 9 |
| 531 | 777 | 2535 | 0 /10 | 0 / 2 | 0 / 9 |
| 531 | 767 | 2545 | 0 /10 | 0 / 2 | 0 / 9 |
| 606 | -3020 | 2552 | 0 /12 | 0 / 5 | 0 / 9 |
| 531 | 736 | 2576 | 0 /10 | 0 / 2 | 0 / 9 |
| 531 | 467 | 2845 | 0 /10 | 0 / 2 | 0 / 9 |
| 606 | 2653 | 3121 | 0 /12 | 0 / 5 | 0 / 9 |
| 531 | -44 | 3356 | 0 /10 | 0 / 2 | 0 / 9 |
| 606 | 2957 | 3425 | 0 /12 | 0 / 5 | 0 / 9 |
| 606 | 3148 | 3616 | 0 /12 | 0 / 5 | 0 / 9 |

^a^ Converted allele (selected site).

^b^ Position of polymorphic sites in *ry^531^* and *ry^606^*, listed in increasing distance. See Table S2 for description of sites.

^c^ Distance from converted allele to unselected site, in base pairs.

^d^ Number of co-conversions and number of total conversions for wild type (w.t.) at room temperature (RT) and 25° C and in *rec* mutants.
